# Geometrically Nonlinear Field Fracture Mechanics and Crack Nucleation, Application to Strain Localization Fields in Al-Cu-Li Aerospace Alloys

**DOI:** 10.3390/ma11040498

**Published:** 2018-03-27

**Authors:** Satyapriya Gupta, Vincent Taupin, Claude Fressengeas, Mohamad Jrad

**Affiliations:** 1 Université de Lorraine, CNRS, Arts et Métiers ParisTech, LEM3, F-57000 Metz, France; satyapriya.gupta@univ-lorraine.fr (S.G.); claude.fressengeas@univ-lorraine.fr (C.F.); mohamad.jrad@univ-lorraine.fr (M.J.); 2 Laboratory of Excellence on Design of Alloy Metals for low-mAss Structures (DAMAS), Université de Lorraine, Nancy-Metz, France

**Keywords:** disconnection density, displacement discontinuity, crack nucleation, crack opening displacement, digital image correlation, Al-Cu-Li alloys

## Abstract

The displacement discontinuity arising between crack surfaces is assigned to smooth densities of crystal defects referred to as disconnections, through the incompatibility of the distortion tensor. In a dual way, the disconnections are defined as line defects terminating surfaces where the displacement encounters a discontinuity. A conservation statement for the crack opening displacement provides a framework for disconnection dynamics in the form of transport laws. A similar methodology applied to the discontinuity of the plastic displacement due to dislocations results in the concurrent involvement of dislocation densities in the analysis. Non-linearity of the geometrical setting is assumed for defining the elastic distortion incompatibility in the presence of both dislocations and disconnections, as well as for their transport. Crack nucleation in the presence of thermally-activated fluctuations of the atomic order is shown to derive from this nonlinearity in elastic brittle materials, without any algorithmic rule or ad hoc material parameter. Digital image correlation techniques applied to the analysis of tensile tests on ductile Al-Cu-Li samples further demonstrate the ability of the disconnection density concept to capture crack nucleation and relate strain localization bands to consistent disconnection fields and to the eventual occurrence of complex and combined crack modes in these alloys.

## 1. Introduction

Earlier work devoted to the applications of differential geometry to fracture processes have suggested that cracks in solids could be modeled by dislocations [1,2,3,4,5,6]. Indeed, several authors used fictitious dislocation fields as mathematical tools for generating the stress-field around cracks in elastic and elasto-plastic materials [7,8,9,10,11]. However, it is well known that dislocations reflect the discontinuity of the elastic/plastic displacement field across some bounded surface in a continuum [12], whereas fracture results in the discontinuity of the total displacement field. Thus, the dislocations involved in the calculations do not actually describe the disruption of matter inherent to fracture. Line defects distinct from dislocations, and referred to as disconnections, have been introduced to consistently account for such discontinuity [13]. Typically, the topological feature associated with the disconnection field is the crack opening displacement (COD), a quantity clearly distinct from the Burgers vector associated with dislocation fields. The approach is grounded on the assignment of the displacement discontinuity across the crack surfaces to a smooth field of incompatible distortion tensors by using the Stokes theorem. A similar procedure applied to the plastic displacement discontinuity in the presence of dislocations results in concurrently involving the incompatibility of the plastic distortion and dislocation densities in the analysis. As a consequence, the elastic distortion field has to accommodate the plastic and total incompatibilities concurrently arising from both dislocations and disconnections. Due to smoothness of this field, the stress field is continuous at all points, including in the dislocation core and crack tip areas.

From a dynamic perspective, large stresses almost invariably result in some dislocation motion in the crack tip area in crystalline materials. Thus, plastic relaxation of the elastic stress field should accompany the elastic unloading due to crack growth, and plastic dissipation in the body should result concurrently from crack growth and dislocation motion. Therefore, crack growth is bound to occur if sufficient energy is available for sustaining both mechanisms, and identifying the driving force for crack growth requires a global approach to dissipation, also involving dislocation motion. After other approaches [10,14,15,16], such a framework was provided in [13] by using a transport scheme for the dislocation and disconnection density tensors: crack growth occurs through disconnection transport, just as plasticity occurs through dislocation transport. The driving force for disconnection motion (and hence for crack growth) is dual to the disconnection velocity in the volumetric dissipation density, in analogy with the Peach–Koehler force on dislocations. Hence, the thermodynamic requirement of non-negative dissipation can be used to formulate guidelines for the constitutive relationship between the driving force for crack growth and the disconnection velocity. As such, the theory provides a dynamic framework for crack growth, and it appears to be well suited for investigating the interplay between crack propagation and plasticity. The role of rotational incompatibility in the stress field of cracks and the influence of rotational defects on plasticity and crack growth was further detailed in [17] for a more accurate investigation of nano-sized neighborhoods of the crack tips. In the present paper, a geometrically nonlinear setting is proposed for the determination of the stress field in the presence of disconnections and dislocations, as well as for their transport scheme, which extends the work of [13] to finite transformations. The implications of this nonlinearity in the modeling of crack nucleation and growth are investigated in elastic-brittle materials. Further insights into the Griffith-based thermally-activated model of crack nucleation [18,19,20] will result from this approach.

Finally, because the present framework is able to relate the discontinuity of the material displacement in forming cracks to the incompatibility of the total elastic plus plastic distortion field, we think that it can be of practical use to understand complex links between strain localization and fracture in ductile materials. In particular, it can be applied to experimental strain fields, for instance to those obtained as per well-used digital image correlation (DIC) methods [21]. In this paper, we indeed consider DIC methods to investigate particular Al-Cu-Li aerospace alloys where complex interactions between localized plasticity and damage evolution leads to failure, and thus, they are interesting for our proposed framework. Indeed, as shown by recent studies on such alloys [22,23], the latter are prone to early and intermittent strain localization followed by fracture within bands. In an Al-Cu-Mg alloy for instance [23], tearing experiments combined with in situ synchrotron laminography and digital volume correlation showed the occurrence of early and intermittent localization bands, certainly associated with dynamic strain aging. Fracture eventually takes place along these bands from damage induced by strain localization. Therefore, we anticipate that, by integrating in time and space the evolution of disconnection densities during deformation, the eventual occurrence of fracture can be related to the accumulation of strain heterogeneity and incompatibility. The Al-Cu-Li alloys can thus be seen as a model benchmark material for validating our disconnection framework. As will be shown indeed, the disconnection density concept is able to capture crack nucleation and to relate complex strain localization features to the crack configurations, which validates the present framework.

The paper is organized as follows. Notations are settled in Section 2. Section 3 includes the fundamental basis for a field theory of the incompatibility of the lattice distortion and its relations, through elasticity, with the plastic incompatibility arising from the presence of dislocations, in a geometrically nonlinear setting. In Section 4, the solution of the boundary value problem for an elasto-static body containing a distribution of disconnection and dislocation densities is provided. Transport of the disconnection and dislocation densities is presented in Section 5, also in a geometrically nonlinear setting. Several algorithms for the solution of boundary value problems are presented in Section 6. The implications of the theory on the interpretation of crack nucleation phenomena are shown in Section 7 by modeling nucleation in elastic-brittle materials. The application of the framework to DIC strain fields and to the description of failure in ductile Al-Cu-Li alloys follows.

## 2. Notations

A bold symbol denotes a tensor. When there may be ambiguity, an arrow is superposed to represent a vector: $\vec{V}$. The symmetric part of the second-order tensor A is denoted $A^{sym}$, and its transpose is $A^{t}$. The tensor A.B, with rectangular Cartesian components $A_{ik}B_{kj}$, results from the dot product of tensors A and B, and A⊗B is their tensorial product, with components $A_{ij}B_{kl}$. A :represents the inner product of the two second-order tensors $A:B=A_{ij}B_{ij}$, in rectangular Cartesian components, or the product of a higher order tensor with a second-order tensor, e.g., ${\left(A:B\right)}_{ij}=A_{ijkl}B_{kl}$. The cross product of a second-order tensor A and a vector V, the div and curl operations for second-order tensors are defined row by row, in analogy with the vectorial case. For any base vector $e_{i}$ of the reference frame:

(1) $$\begin{array}{ccc} {\left(A\times V\right)}^{t}.e_{i} & = & (A^{t}.e_{i})\times V \end{array}$$

(2) $$\begin{array}{ccc} {\left(\mathrm{div}\;A\right)}^{t}.e_{i} & = & \mathrm{div}(A^{t}.e_{i}) \end{array}$$

(3) $$\begin{array}{ccc} {\left(\mathrm{curl}\;A\right)}^{t}.e_{i} & = & \mathrm{curl}(A^{t}.e_{i}). \end{array}$$

In rectangular Cartesian components:

(4) $$\begin{array}{ccc} {\left(A\times V\right)}_{ij} & = & e_{jkl}A_{ik}V_{l} \end{array}$$

(5) $$\begin{array}{ccc} {\left(\mathrm{div}A\right)}_{i} & = & A_{ij,j} \end{array}$$

(6) $$\begin{array}{ccc} {\left(\mathrm{curl}A\right)}_{ij} & = & e_{jkl}A_{il,k}. \end{array}$$

where $e_{jkl}$ is a component of the third-order alternating Levi–Civita tensor X. A vector $\vec{A}$ is associated with tensor A by using its inner product with tensor X:

(7) $$\begin{array}{c} {\left(\vec{A}\right)}_{k}=-\frac{1}{2}{\left(A:X\right)}_{k}=-\frac{1}{2}e_{ijk}A_{ij}. \end{array}$$

A superposed dot represents a material time derivative. In the component representation, the spatial derivative with respect to a Cartesian coordinate is indicated by a comma followed by the component index.

## 3. Incompatible Elasto-Plastic Continuum Theory

### 3.1. Disconnections and the Incompatibility of the Total Distortion

In the framework of field models, the displacement vector u is usually defined continuously at any point of a body B undergoing elasto-plastic transformation. If X and x are respectively the reference (Lagrange) and current (Euler) position vectors of a material element, the transformation tensor F is defined as the Jacobian tensor ∂x/∂X. The gradient of the displacement is:

(8) U=F−I=gradu,

where I is the identity tensor. As such, U is curl-free:

(9) curlU=0,

a condition also verified by F: curlF=0. Equation (9) is a necessary condition for the integrability of the distortion U, or equivalently for finding a single-valued displacement field u from Equation (8). It is usually referred to as a compatibility condition for U. The distortion tensor may be decomposed into its symmetric part, i.e., the strain tensor $\epsilon =U^{sym}$, and its skew-symmetric part, the rotation tensor $\omega =U^{skew}$, or equivalently the rotation vector:

(10) $$\vec{\omega }=\frac{1}{2}\;\mathrm{curl}\;u.$$

From Equation (10), it is seen that a necessary condition for the integrability of the rotation is:

(11) $$\mathrm{div}\;\vec{\omega }=0,$$

and that the compatibility condition (9) also reads:

(12) $$\mathrm{curl}\epsilon -{\mathrm{grad}}^{t}\vec{\omega }=0.$$

Transposing Equation (12) and taking the curl of the result provides a necessary condition on the integrability of the strain tensor known as Saint-Venant’s compatibility relation:

(13) $${\mathrm{curl}\;\mathrm{curl}}^{t}\;\epsilon =0.$$

**Figure 1:**
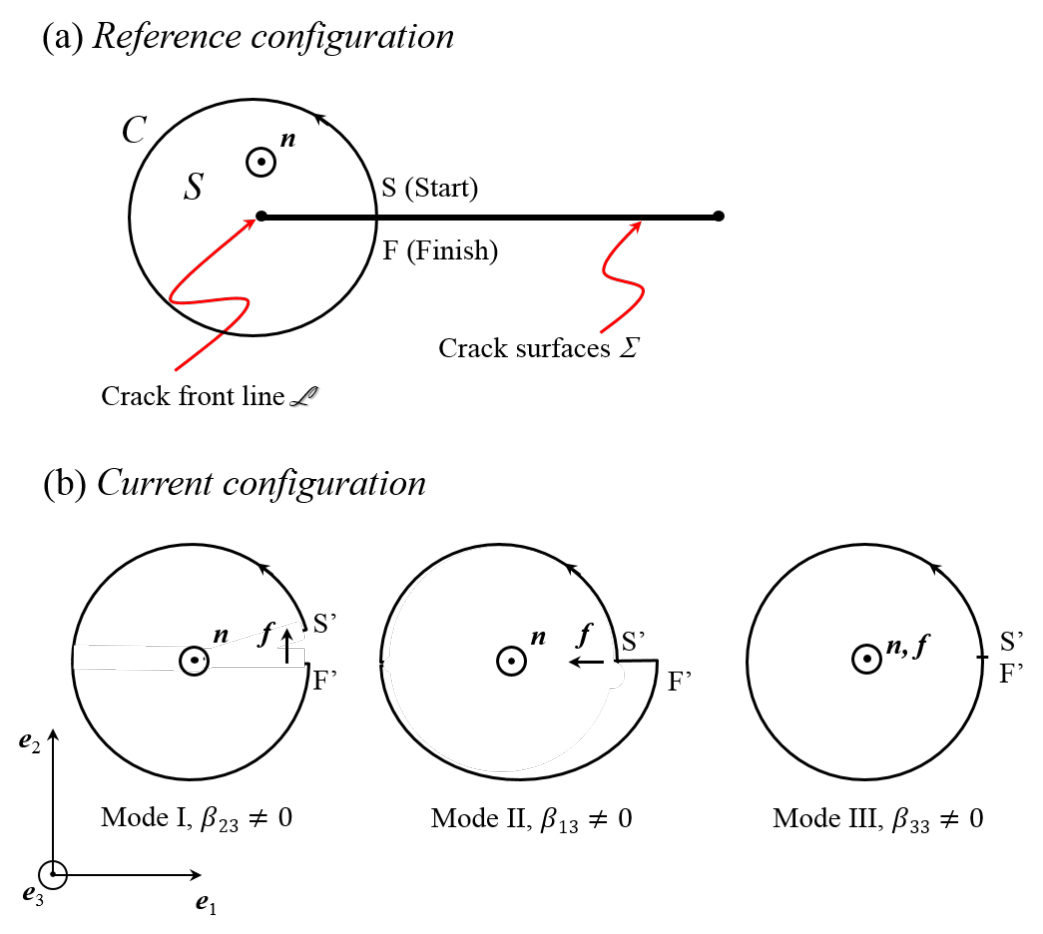
Crack surfaces Σ and front line L edge-on, test surface *S* with unit normal n and oriented bounding circuit *C*; (**a**) reference configuration, points (S,F) coincide; (**b**) current configuration with crack opening displacement (COD): $f=F^{\prime }S^{\prime }$ and fracture Modes I, II and III.

Conversely, taken with Equation (11), Equation (13) is sufficient to insure the existence of a single-valued continuous solution u to Equation (8), up to a constant translation. More directly, Equation (9) is also a sufficient condition for the existence of this displacement field. However, displacement discontinuities may occur across bounded surfaces Σ, and the displacement field may cease to be single-valued if cracks nucleate and develop along such surfaces. A closed circuit *C* drawn on the body in the reference configuration and threading once the surface Σ may therefore present a closure defect f in the current configuration (see Figure 1). A definition for f is:

(14) $$\begin{array}{ccc} f & = & -\int_{C}F.\mathrm{dX}=-\int_{C}U.\mathrm{dX}=-\int_{S}\mathrm{curl}\;U.ndS \end{array}$$

if the following convention is adopted: the circuit *C* is oriented clockwise; the starting point *S* and finishing point *F* coincide in the reference configuration; and the closure defect is $f=F^{\prime }S^{\prime }$ in the current configuration, where $S^{\prime }$ and $F^{\prime }$ denote the transforms of points *S* and *F*. In this relation, the surface *S*, with unit normal n in the reference configuration, is bounded by curve *C*. f actually characterizes a line defect: it is the displacement discontinuity occurring across the surface Σ. Let us denote by L the closed line bounding Σ and l its tangent vector. L threads surface *S* and is the front line of the disconnected crack surfaces in the current configuration. The displacement discontinuity f is usually referred to as the crack opening displacement (COD). Note that the possibility of a discontinuity of the rotation field is disregarded in the present paper, which limits the accuracy of the analysis in the close vicinity of the front line L. This issue was further investigated in [17] in relation to the development of cracks in nanograined materials. Using the Stokes theorem (The Stokes theorem applies in simply connected bodies, and more generally in all simply connected parts of a multiply-connected body. It is simply required that the complete boundary curve be involved in the line integral, with adequate orientation.), the above displacement incompatibility can be reflected in an average manner over the surface *S* by the continuous tensorial density β:

(15) $$\begin{array}{ccc} \beta & = & -\mathrm{curl}\;U. \end{array}$$

Of course, this equation still holds if U is replaced with F. We shall refer to β as the disconnection density tensor (After J.P.Hirth [3], the word “disconnection” has been associated with interfacial defects of dislocation and/or step character in relation to grain boundary migration or twinning/phase transformation mechanisms through interface propagation. Of course, it carries a different meaning in the present context. “Disconnection” presently reflects broken atomic bonds and a locally disconnected material. Beyond being consistent with physical intuition, this usage is in agreement with mathematical terminology. Indeed, if the incipient crack tunnels from side to side through the body, the topological status of the latter shifts from simply connected to multiply connected.). Clearly, the Saint-Venant’s compatibility condition (13) is not satisfied if β is a non-vanishing tensor. Taking the same steps as above, it can indeed be shown that the tensor $\eta \;=\;{\mathrm{curl}\;\mathrm{curl}}^{t}\;\epsilon$ is now:

(16) $$\eta =-\mathrm{curl}\beta^{t}.$$

η may be referred to as an incompatibility tensor. The geometrical meaning of Equation (16) is that the continuity of matter is disrupted if the incompatibility tensor η is non-vanishing in the presence of disconnections β. According to Equation (14), β is also the dyadic product:

(17) β=f⊗n

for a unit surface of normal n. If a disconnection ensemble of line vector l and crack opening displacement $\tilde{f}$ threads the unit surface of normal l, then only the part l.n of this ensemble threads the unit surface of normal n. The corresponding crack opening displacement is $f=\left(l.n\right)\tilde{f}$, and from Equation (17):

(18) $$\beta =\left(l.n\right)\tilde{f}⊗n=\tilde{f}⊗l.$$

The dimension of the disconnection tensor components is m${}^{-1}$, i.e., *m* of displacement discontinuity per m${}^{2}$. Equation (18) represents a Mode I or Mode II fracture when $\tilde{f}.l=0$, whereas a Mode III crack is reflected if $\tilde{f}\times l=0$. For instance, if a planar crack is considered as in Figure 1, with $e_{2}$ and $e_{3}$ as the unit normal to the fracture plane and the rectilinear fracture line direction respectively in the orthogonal frame $\left(e_{1},e_{2},e_{3}\right)$, we may refer to the components $\beta_{23}$ and $\beta_{13}$ as edge-disconnection densities associated with Mode I and Mode II, respectively, and to $\beta_{33}$ as a screw-disconnection density associated with Mode III fracture. The emergence of an edge-disconnection density field in relation to crack nucleation is illustrated below by using DIC records of tensile tests on Al-Cu-Li samples in Section 7. From Equation (15), it is seen that β necessarily satisfies the relation:

(19) divβ=0,

which has the meaning that disconnection lines cannot end within the body. If they do not exit the body, these lines are indeed loops terminating surfaces of discontinuity of the displacement field.

Further insights into the distortion incompatibility deriving from the presence of disconnections are provided by its Stokes–Helmholtz orthogonal decomposition. Invoking the latter in the space of square-integrable tensor fields with square-integrable first order derivatives (see for example [24], Theorem 5.8), there exists indeed a unique tensor field χ and a unique (up to a constant) vector field z (both square-integrable, as well as their derivatives to second-order) such that the distortion field U reads as the sum:

(20) U=curlχ+gradz.

with the orthogonality condition $\int_{D}\mathrm{curl}\;\chi :\mathrm{grad}\;z\;dv=0$. Thus, taking the curl of U in Equation (20) extracts curlχ and discards gradz, whereas divU extracts gradz and eliminates curlχ. Therefore, Equation (15) involves only curlχ, which will be identified below as the incompatible part $U^{⊥}$ of U:

(21) $$\begin{array}{ccc} {\mathrm{curl}\;U}^{⊥} & = & \mathrm{curl}\;\mathrm{curl}\;\chi =-\beta . \end{array}$$

Similarly, gradz will be the compatible part $U^{∥}$ of the distortion U. Up to a constant, z will be the compatible part of the displacement, $u^{∥}$. To ensure correctness of this identification, $U^{⊥}$ must vanish identically throughout the body when β=0. Thus, following [24], Equation (21) is augmented with the side conditions $\mathrm{div}\;U^{⊥}=0$ and $U^{⊥}.n=0$ on the external boundary with unit normal n. Then, any residual gradient part gradw of $U^{⊥}$ satisfies a Poisson equation divgradw=0, and the boundary condition gradw.n=0 guarantees a vanishing w. As a consequence, the incompatible distortion field $U^{⊥}$ is uniquely determined once the disconnection density field is known. The incompatible part of the displacement, $u^{⊥}$, which is to be identified with the crack opening displacement, follows from Equations (14), (15) and (21) through an integral on a patch *S*:

(22) $$u^{⊥}=f=\int_{S}\beta .ndS.$$

The compatible displacement, $u^{∥}$, and the compatible part $U^{∥}$ of the distortion tensor are used for calculating the Jacobian *J*, i.e., the determinant of the tensor ∂x/∂X, in the statement of mass conservation $\rho J=\rho_{0}$, where $\rho_{0}$ and ρ are respectively the initial and current mass densities.

### 3.2. Dislocations and the Incompatibility of the Plastic Distortion

When dislocations are present in the body B, a closed circuit *C* drawn in the reference configuration may also present a closure defect reflecting a discontinuity of the plastic displacement, referred to as the Burgers vector b, in the intermediate local stress-free configuration wherein the body is not elastically distorted. Consistent with earlier assumptions in the present paper, the possibility of a discontinuity of the plastic rotation is disregarded in the present paper. Acknowledging this possibility would amount to considering the presence of disclinations [17,25]. Using the same sign convention as in Equation (14), a definition for b is:

(23) $$\begin{array}{ccc} b & = & -\int_{C}F_{p}.\mathrm{dX}=-\int_{S}\mathrm{curl}\;F_{p}.ndS=-\int_{S}\mathrm{curl}\;U_{p}.ndS \end{array}$$

where $F_{p}$ is the plastic transformation tensor in the elastic/plastic multiplicative decomposition $F\;=\;F_{e}.F_{p}$ of the transformation tensor [26] and $U_{p}=F_{p}-I$ is the corresponding plastic distortion. As argued above for the disconnection and total distortion tensors, Equation (23) can be used to define Nye’s dislocation density tensor α by exchanging the incompatibility of the smooth plastic distortion tensor for the discontinuity b of the plastic displacement:

(24) $$\begin{array}{ccc} {\mathrm{curl}\;U}_{p} & = & -\alpha . \end{array}$$

Invoking again the Stokes–Helmholtz decomposition of $U_{p}$, there exist a gradient-free incompatible part of the plastic distortion, $U_{p}^{⊥}$, such that:

(25) $$\begin{array}{ccc} {\mathrm{curl}\;U}_{p}^{⊥} & = & -\alpha . \end{array}$$

Again, the same equation holds if $U_{p}^{⊥}$ is replaced with $F_{p}^{⊥}$. $U_{p}^{⊥}$ is uniquely determined from the dislocation density field α if it is demanded that it also satisfies the side conditions ${\mathrm{div}\;U}_{p}^{⊥}=0$ and $U_{p}^{⊥}.n=0$ on the external boundary with unit normal n. The compatible part $U_{p}^{∥}$ of the plastic distortion contributes to the accumulated plastic strain, but does not play a role in the mechanical state of the material or in the definition of the dislocation density α. From Equation (25), it is additionally seen that:

(26) divα=0,

which means that dislocation lines cannot end within the body. Although α is carrying a topological content b reflecting the distortion of the material in the intermediate configuration, it operates on lines t in the reference configuration. In its standard definition, Nye’s dislocation density tensor operates on lines in the current configuration, and produces Burgers vectors in the local intermediate configuration [12]. Thus, in the present work, the components $\alpha_{ij}$ of Nye’s tensor are dislocation line densities with respect to surface units in the reference configuration, whereas they are referred to surface units in the current configuration in the standard definition. These two dislocation density measures may differ significantly at finite strains, as detailed below in Equations (30) and (31). However, the Burgers vectors obtained from both definitions are strictly identical. Further, the present definition of the dislocation density tensor is natural from a physical perspective, because dislocations are the cause of the incompatible plastic distortion, whereas the incompatible elastic distortion derives from the presence of both dislocations and disconnections. In the presence of dislocations, it is such that either it maintains the continuity of matter in the absence of disconnections, or it also accommodates the total distortion incompatibility in their presence. The derivation of the elastic distortion incompatibility will be the subject of the next section. The formal similarity in the treatment of dislocation and disconnection densities shown above should not hide their fundamental topological difference. Dislocations reflect a discontinuity of the plastic displacement field across the surface they sweep, but continuity of matter is maintained along this surface. Conversely, disconnections disrupt the continuity of matter along their paths, whose evidence therefore remain tangible in the body.

### 3.3. Composition of Incompatibilities and Elastic Distortion

Because the incompatibilities of the total and plastic distortion fields both impact on the elastic state of the body, they must compose and the elastic transformation tensor $F_{e}$ of the lattice must adjust to the dislocation and disconnection density fields. Taking the curl of the multiplicative decomposition $F=F_{e}.F_{p}$, we obtain successively:

$$\begin{array}{ccc} {\left(\mathrm{curl}\;F\right)}_{ij} & = & e_{jkl}{\left(F_{im}^{e}F_{ml}^{p}\right)}_{,k}=F_{im}^{e}e_{jkl}F_{ml,k}^{p}+e_{jkl}F_{im,k}^{e}F_{ml}^{p}\\ {\left(\mathrm{curl}\;F\right)}_{ij} & = & F_{im}^{e}{\left({\mathrm{curl}\;F}_{p}\right)}_{mj}+e_{jkl}G_{imk}^{e}F_{ml}^{p}\\ {\left(\mathrm{curl}\;F\right)}_{ij} & = & {\left(F_{e}.{\mathrm{curl}\;F}_{p}\right)}_{ij}+e_{jkl}G_{ikm}^{e,T}F_{ml}^{p}, \end{array}$$

which translates into the intrinsic relation:

(27) $$\mathrm{curl}\;F=F_{e}.{\mathrm{curl}\;F}_{p}+G_{e}^{T}.F_{p}:X,$$

where $G_{e}^{T}$ is defined from the second-order elastic distortion $G_{e}$ with components $G_{imk}^{e}=F_{im,k}^{e}$ by transposing the last two subscripts. Substituting Equations (15) and (24) in (27) then yields the differential equation:

(28) $$\beta =F_{e}.\alpha -G_{e}^{T}.F_{p}:X$$

for the elastic distortion $F_{e}$, where the defect densities (α,β) and plastic transformation tensor $F_{p}$ are prescribed quantities. In this form, the solution $F_{e}$ is not unambiguously defined because the compatible part $F_{p}^{∥}$ of $F_{p}$ arbitrarily depends on the choice of the reference configuration. Hence, we choose $F_{p}^{∥}=0$ with no loss of generality, and transform Equation (28) into:

(29) $$\beta =F_{e}.\alpha -G_{e}^{T}.F_{p}^{⊥}:X$$

where $F_{p}^{⊥}$ derives from the disconnection density field β through the solution of Equation (25). Therefore, $F_{e}$ can be found uniquely from Equation (29) once the fields (α,β) are known and boundary conditions are prescribed. This issue will be dealt with in the next section. In the absence of disconnections (β=0), $F_{p}^{⊥}=F_{e}^{-1}$, and Equation (29) leads to the relation:

(30) $$\alpha =F_{e}^{-1}.G_{e}^{T}.F_{e}^{-1}:X.$$

The dislocation density tensor found in Equation (30) differs from the conventional definition of the dislocation density tensor in the absence of disconnections:

(31) $$\alpha =-\mathrm{curl}\;F_{e}^{-1},$$

because, as already mentioned, it operates on surface elements in the reference configuration, in place of the current configuration. Of course, by restricting the purpose to small transformations, it is seen that Equation (29) becomes successively:

(32) $$\begin{array}{ccc} \beta_{ij} & ≅ & \alpha_{ij}-U_{im,k}^{e}\delta_{ml}e_{klj} \end{array}$$

(33) $$\begin{array}{ccc} \beta_{ij} & ≅ & \alpha_{ij}-e_{jkl}U_{il,k}^{e} \end{array}$$

(34) $$\begin{array}{ccc} {\mathrm{curl}\;U}_{e}^{⊥} & ≅ & \alpha -\beta , \end{array}$$

which coincides with the composition relation provided in [13] and, when β=0, with the approximation of Equation (31) at small distortions:

(35) $$\alpha =\mathrm{curl}\;U_{e}^{⊥}.$$

Equations (29) and (34) suggest that the (α,β) tensor fields may be simultaneously non-zero at a given material point, implying that the present description intrinsically belongs to the cohesive approaches of fracture. Indeed, (α,β) would be mutually exclusive in a non-cohesive description of fracture: (α≠0,β=0) or (α=0,β≠0). In the geometrically linear context, Equation (34) suggests that a given incompatible elastic distortion field can be obtained from either a distribution of dislocations or the same distribution of disconnections (with changed sign). However, such an identification does not imply equality of the associated stress fields, as zero-traction boundary conditions need to be fulfilled on the crack surfaces in the case of disconnections. In the geometrically nonlinear case, it appears rather that the dislocation and disconnection density fields do not play such a symmetric role in the stress field. Changing the sign of the dislocation distribution in Equation (29) is not equivalent to introducing a disconnection density field. Further details on calculating the stress field associated with a distribution of dislocations and disconnections in this cohesive modeling scheme are provided in the following Section.

## 4. Elasto-Static Incompatible Media

We assume that the body B contains a distribution of dislocations α and disconnections β. Provisionally, we are not interested in the dynamic plastic and fracture processes leading to this distribution, and the history-dependent compatible part of the plastic transformation, $F_{p}^{∥}$, and plastic displacement, $u_{p}^{∥}$, are arbitrarily set to zero without loss of generality. The body is in equilibrium under traction loads $F_{\partial B_{t}}\left(P\right)$ applied at points *P* on a part $\partial B_{t}$ of its external surface ∂B, including in particular the non-cohesive parts of the crack surfaces $\partial B_{c}$ where $F_{\partial B_{t}}\left(P\right)=0$, and other loads leading to prescribed displacements u(P) on the remaining part $\partial B_{u}$ of the external surface. The following equations are therefore satisfied:

(36) $$\begin{array}{ccc} G_{e}^{T}.F_{p}^{⊥}:X & = & F_{e}.\alpha -\beta \end{array}$$

(37) $$\begin{array}{ccc} T & = & T(F_{e}) \end{array}$$

(38) $$\begin{array}{ccc} \mathrm{div}\;T & = & 0. \end{array}$$

In this set of equations, geometric nonlinearity is assumed, and Equation (36) is reproduced after Equation (29). The stress tensor T is provided as a function of the elastic transformation tensor $F_{e}$, for instance through the right Cauchy–Green tensor $F_{e}^{t}.F_{e}$. In the balance of momentum (38), the inertial terms and volumetric load density are neglected as being unessential in the present context. As a first step of the solution process, an incompatible solution $F_{e}^{⊥}$ of Equation (36) is searched for by appending to the latter the side conditions:

(39) $$\begin{array}{ccc} \mathrm{div}\;F_{e} & = & 0 \end{array}$$

(40) $$\begin{array}{ccc} F_{e}.n & = & 0\;\;\mathrm{on}\;\partial \;B. \end{array}$$

As already explained in Section 3.1, the conditions (39),(40) guarantee that $F_{e}$ does not have a gradient component and is therefore incompatible. Generally, the field $F_{e}^{⊥}$ does not satisfy the balance of momentum equation, and we consequently look for the complementary compatible transformation $F_{e}^{∥}$ such that the total elastic transformation $F_{e}$ satisfies Equations (37) and (38) under the boundary conditions detailed above. In the present nonlinear context, it is not possible to prove the existence and uniqueness of such a solution, but in a simplified version where the material response is assumed to be linear, it can be shown that such a solution does exist. Let indeed the elastic response be specified as:

(41) $$T=C:U_{e}^{sym}=C:\epsilon_{e}$$

where C is the fourth-order tensor of elastic moduli and $\epsilon_{e}$ is the symmetric part of the elastic distortion tensor, i.e., the elastic strain tensor. Separating the compatible and incompatible parts of the latter:

(42) $$\epsilon_{e}=\epsilon_{e}^{⊥}+\epsilon_{e}^{∥},$$

and combining Equations (38), (41) and (42), we find:

(43) $$\mathrm{div}\;\left(C:\epsilon_{e}^{∥}\right)+f^{⊥}=0,\;f^{⊥}=\mathrm{div}\;\left(C:\epsilon_{e}^{⊥}\right)$$

where $f^{⊥}$ is a volumetric density of loads reflecting the stress field $C:\epsilon_{e}^{⊥}$ that arises in the body from the presence of the dislocation and disconnection fields (α,β). In addition to Equation (43), the compatible elastic strain $\epsilon_{e}^{∥}$ and corresponding elastic displacement $u_{e}^{∥}$ must also satisfy the boundary conditions:

(44) $$\begin{array}{ccc} u_{e}^{∥}\left(P\right) & = & u\left(P\right)-u_{e}^{⊥}\left(P\right)-u^{⊥}\left(P\right),\;\forall P\in \partial B_{u} \end{array}$$

(45) $$\begin{array}{ccc} C:\epsilon_{e}^{∥}.n & = & F_{\partial D_{t}}\left(P\right)-C:\epsilon_{e}^{⊥}.n,\;\forall P\in \partial B_{t} \end{array}$$

where $u_{e}^{⊥}\left(P\right)$ results from the incompatible elastic distortion $U_{e}^{⊥}$ and $u^{⊥}\left(P\right)$ is found from Equation (22) through an integral on a patch *S* around point *P*:

(46) $$\forall P\in B,u^{⊥}\left(P\right)=\int_{S}\beta .ndS.$$

For given dislocation and disconnection densities (α,β), this is a conventional Navier-type elasticity problem, whose solution, i.e., the elastic displacement field $u_{e}^{∥}$, is unique. Once the displacement is arbitrarily chosen at some point $P_{0}\in \partial B_{u}$ where it is uniquely defined, the compatible displacement field at any point P∈D is computed from the path-integral:

(47) $$\forall P\in B,u^{∥}\left(P\right)=\int_{P_{0}}^{P}U_{e}^{∥}.\mathrm{dX}+u\left(P_{0}\right)$$

Clearly, the integral (47) is path-dependent in the presence of dislocations and disconnections.

## 5. Transport

Because the dislocation and disconnection density tensors are areal densities of lines carrying a topological content (the Burgers and COD vectors), their evolution is governed by statements for the conservation of this content. We assume that the dislocation and the disconnection densities have associated velocities with respect to the material, $V_{\alpha }$ and $V_{\beta }$ respectively. When the resolution length scale is small enough, these density fields represent individual defects, and the corresponding values of the velocity fields represent the motion and deformation of the core regions of the defects. When, instead, the resolution length scale is sufficiently large, the surface *S* bounded by circuit *C* in Equations (14) and (23) may be threaded by several defect lines. Then, the velocity fields reflect the collective motion of these lines with respect to the material. Their advance represents the spreading of one topologically-charged piece of the body at the expense of the other. Let us consider an element of a disconnection ensemble, of line vector $t_{0}$ in the reference configuration $B_{0}$ of the body B and COD f per unit surface of the current configuration $B_{t}$, having a velocity $V_{\beta }$ with respect to the lattice in this configuration. The disconnection density tensor associated with this disconnection element is $\beta =f⊗t_{0}$. In its motion, this disconnection element crosses an oriented closed curve $C_{0}$ drawn in the initial configuration $B_{0}$ and enters (or exits) the surface $S_{0}$ bounded by $C_{0}$. Thus, some COD flux F is generated through the differential element ${\mathrm{dx}}_{0}$ tangent to curve $C_{0}$, and the rate of CODs through the surface ${\mathrm{dS}}_{0}=t_{0}\times {\mathrm{dx}}_{0}$ is:

(48) $$F.{\mathrm{dx}}_{0}=f\left(V_{\beta }^{0}.{\mathrm{dS}}_{0}\right)=f\left(V_{\beta }^{0}.\left(t_{0}\times {\mathrm{dx}}_{0}\right)\right),$$

where $V_{\beta }^{0}$ is the disconnection velocity with respect to the reference frame. Composing with the material velocity v with respect to the reference frame, the latter is also $V_{\beta }^{0}=v+V_{\beta }$. Using a permutation of the mixed product allows extracting: ${\mathrm{dx}}_{0}$

(49) $$F.{\mathrm{dx}}_{0}=-f\left(\left(t_{0}\times V_{\beta }^{0}\right).{\mathrm{dx}}_{0}\right)=-f⊗\left(t_{0}\times V_{\beta }^{0}\right).{\mathrm{dx}}_{0},$$

which leads to the identification of the COD flux tensor as:

(50) $$F=-\left(f⊗t_{0}\right)\times V_{\beta }^{0}=-\beta \times V_{\beta }^{0}=-\dot{U}.$$

Hence, F appears as being opposed to the distortion rate tensor $\dot{U}$. The evolution of the fracture line is typically characterized by $\dot{U}$. Indeed, assume that the resolution length scale is small enough to envision a single defect, and consider a planar crack in the orthonormal basis $\left(e_{1},e_{2},e_{3}\right)$ with $e_{2}$ and $e_{3}$ as the unit normal to the fracture plane and the rectilinear crack line direction respectively. If $V_{\beta }^{0}=V_{1}e_{1}+V_{2}e_{2}$ is the crack line velocity, then it is found from Equation (50) that $\dot{U}.e_{2}=\beta_{23}V_{1}e_{2}$ in Mode I for the disconnection tensor $\beta =\beta_{23}e_{2}⊗e_{3}$, $\dot{U}.e_{2}=\beta_{13}V_{1}e_{1}$ in Mode II for $\beta =\beta_{13}e_{1}⊗e_{3}$, and in Mode III: $\dot{U}.e_{2}=\beta_{33}V_{1}e_{3}$ for $\beta =\beta_{33}e_{3}⊗e_{3}$, as could be expected. Indeed, the distortion rate induces crack opening by tension, shear perpendicular to the crack line and shear parallel to the crack line in Modes I, II and III, respectively.

It seems reasonable to postulate the following statement of balance: the rate of change of the COD content of the disconnections threading $S_{0}$ is the difference between the incoming and outgoing COD fluxes if no COD is independently generated or absorbed inside $S_{0}$. Employing the COD flux defined in Equation (48), the corresponding balance equation is:

(51) $$\frac{d}{dt}\int_{S_{0}}\beta .n_{0}dS_{0}=\int_{C_{0}}F.{\mathrm{dx}}_{0}.$$

This statement is required to hold for all such patches in the body B. Applying the Stokes theorem to the line integral on the right-hand side and pushing the time derivative inside the integral on the left-hand side, we obtain:

(52) $$\int_{S_{0}}\left(\dot{\beta }-\mathrm{curl}\;F\right).n_{0}dS_{0}=0.$$

For reasons of continuity of the integrand, the equivalent point-wise statement can be extracted:

(53) $$\dot{\beta }-\mathrm{curl}\;F=0$$

at all points in B. Here, $\dot{\beta }$ represents the time derivative of β in the reference frame. Since $S_{0}$ is invariant, Equation (53) is valid for finite transformations. By substituting the relation (50) in Equation (53), this balance statement takes the form:

(54) $$\dot{\beta }+\mathrm{curl}\;\dot{U}=\dot{\beta }+\mathrm{curl}\;\left(\beta \times V_{\beta }^{0}\right)=\dot{\beta }+\mathrm{curl}\;\left(\beta \times \left(v+V_{\beta }\right)\right)=0.$$

Equation (54) may be read as:

(55) $$\dot{\beta }+\mathrm{curl}\;\left(\beta \times V_{\beta }\right)=s_{v},$$

where the term $s_{v}=-\mathrm{curl}\;\left(\beta \times v\right)$ appears as a source for disconnections in the material frame. This source derives from the incompatibility of the distortion rate arising in the motion of the material points supporting the disconnection density. Further analysis of the crack nucleation arising from this source is provided below in Section 7 in the context of elastic-brittle materials. If small transformations can be assumed, the transport Equation (54) simplifies into:

(56) $$\dot{\beta }+\mathrm{curl}\;\left(\beta \times V_{\beta }\right)=0.$$

As for the evolution of the dislocation density field α, it has been shown from the kinematics of flux of lines moving in and out of the area patch *S* through its bounding curve *C* in the current configuration that similar transport equations are obtained for the dislocation densities [27], resulting in:

(57) $$\dot{\alpha }+\mathrm{curl}\;\left(\alpha \times \left(v+V_{\alpha }\right)\right)=0$$

for finite transformations, and:

(58) $$\dot{\alpha }+\mathrm{curl}\;\left(\alpha \times V_{\alpha }\right)=0$$

for small transformations. Similar to Equation (55), it can be stated here that the term $s_{v}=-\mathrm{curl}\;\left(\alpha \times v\right)$ appears as a source for dislocations in the material frame [28]. If suitable constitutive assumptions are made for the motion of dislocations and disconnections, i.e., if the velocities $\left(V_{\alpha },V_{\beta }\right)$ for the transport of dislocation and disconnection densities are specified in terms of appropriate driving forces, then Equations (54), (56)–(58) may be used as governing equations for the dynamics of dislocations and disconnections. Such constitutive assumptions should in particular confer to the transport scheme the ability to reflect a Griffith-type behavior for fracture growth. In addition, if annihilation is pervasive in the dynamics of dislocations and is conveniently treated by Equations (57) and (58) [29], disconnection annihilation may not be as customary because healing of pre-existing cracks can be difficult in certain materials, such as metals below melting temperature in usual pressure conditions. Such material behavior also needs to be reflected by the constitutive assumptions made for the disconnection velocity $V_{\beta }$ in Equations (54) and (56). These assumptions were discussed in [13], based on thermodynamical arguments originally introduced in [30].

## 6. Elasto-Plastic Incompatible Media

Gathering the above analyses together with results from [13] regarding the driving forces on dislocations and disconnections, the equations of the elasto-plastic boundary value problem with crack propagation can be summarized as follows:

(59) $$\begin{array}{ccc} {\mathrm{curl}\;U}_{p}^{⊥} & = & -\alpha \end{array}$$

(60) $$\begin{array}{ccc} {\mathrm{curl}\;U}^{⊥} & = & -\beta \end{array}$$

(61) $$\begin{array}{ccc} G_{e}^{⊥,T}.F_{p}^{⊥}:X & = & F_{e}^{⊥}.\alpha -\beta \end{array}$$

(62) $$\begin{array}{ccc} \mathrm{div}\;F_{e}^{⊥} & = & 0 \end{array}$$

(63) $$\begin{array}{ccc} T & = & C:(\epsilon_{e}^{∥}+\epsilon_{e}^{⊥}) \end{array}$$

(64) $$\begin{array}{ccc} \mathrm{div}\;T & = & 0 \end{array}$$

(65) $$\begin{array}{ccc} \dot{\alpha }+\mathrm{curl}\;\left(\alpha \times \left(v+V_{\alpha }\right)\right) & = & 0 \end{array}$$

(66) $$\begin{array}{ccc} \dot{\beta }+\mathrm{curl}\;\left(\beta \times \left(v+V_{\beta }\right)\right) & = & 0 \end{array}$$

(67) $$\begin{array}{ccc} V_{\alpha } & = & \frac{1}{B_{\alpha }}T^{t}.\alpha :X \end{array}$$

(68) $$\begin{array}{ccc} V_{\beta } & = & -\frac{1}{B_{\beta }}\;T^{t}.\beta :X \end{array}$$

(69) $$\begin{array}{ccc} {\dot{U}}_{p}^{∥} & = & \alpha \times V_{\alpha }. \end{array}$$

Here, nonlinearity of the geometrical setting is imposed but linearity of elasticity is assumed in Equation (63) for the sake of simplicity. The relations (67) and (68) reflect the dependence of the crystal defect velocities $\left(V_{\alpha },V_{\beta }\right)$ on the respective driving forces [13]. $\left(B_{\alpha },B_{\beta }\right)$ are positive drag parameters through which the physics of dislocation and disconnection motion can be prescribed. In such a framework, climb of dislocations is allowed, as well as pressure-dependence of the plastic distortion rate.

As in Section 4, the boundary conditions are prescribed on the compatible displacement field, $u^{∥}$, and the traction vector at the external surfaces of the body B:

(70) $$\begin{array}{ccc} u^{∥} & = & u\left(P\right)-u^{⊥}\left(P\right),\;\forall P\in \partial B_{u} \end{array}$$

(71) $$\begin{array}{ccc} C:\epsilon_{e}^{∥}.n & = & F_{\partial B_{t}}\left(P\right)-C:\epsilon_{e}^{⊥}.n,\;\forall P\in \partial B_{t}. \end{array}$$

However, Equations (70) and (71) differ substantially from the boundary conditions (44) and (45) in the elasto-static problem, because $u^{∥}$ and $U^{∥}$ now include an evolving plastic part. Boundary conditions on the dislocation density tensor α may be additionally prescribed, particularly on the crack surfaces where α may also vanish in non-cohesive regions, but no boundary condition is imposed on the disconnection density tensor β. The unknowns are the compatible displacement field and the dislocation and disconnection fields.

## 7. Disconnection Nucleation

### Thermally-Activated Crack Nucleation in Elastic-Brittle Materials

The current understanding of crack nucleation and growth is based on ideas first initiated by [18]. Following this original presentation, let us recall that creating a circular crack of radius *l* in a perfect elastic solid under a tensile stress σ perpendicular to the crack costs an amount of energy of the order of $2\gamma \pi l^{2}$, if γ is the energy needed to break atomic bonds and generate new surfaces over a unit area. The stored elastic energy density in the solid being $\sigma^{2}/2E$ with *E* as Young’s modulus, this crack allows releasing the stored elastic energy $-\left(4\pi l^{3}/3\right)\left(\sigma^{2}/2E\right)$ from the volume $4\pi l^{3}/3$. The total energy variation in nucleating a crack of length *l* is therefore:

(72) $$W=2\gamma \pi l^{2}-\frac{2\pi l^{3}\sigma^{2}}{3E}.$$

Crack initiation shall therefore result from a competition between elastic energy relaxation and atomic bond stretching. When *l* is varied, *W* goes through the maximum:

(73) $$W_{m}=\frac{8\pi \gamma^{3}E^{2}}{3\sigma^{4}}$$

for $l=l_{c}=2\gamma E/\sigma^{2}$. Increasing the crack length costs a positive amount of energy when $l<l_{c}$, but releases a negative amount if $l>l_{c}$, which fuels crack growth. The critical condition that the crack may extend therefore occurs for $l=l_{c}$ at the maximum of energy, because crack growth becomes sustained beyond this point. Yet, crack growth may occur when $l<l_{c}$ if thermal fluctuations of the lattice activate the phenomenon. Then, according to the Arrhenius law, the lifetime $t_{N}$ of the undamaged solid is such that:

(74) $$\frac{1}{t_{N}}=\nu_{N}=\nu_{0}exp\left(-\frac{W_{m}}{kT}\right)=\nu_{0}exp\left(-\frac{8\pi \gamma^{3}E^{2}}{3kT\sigma^{4}}\right)$$

where $\nu_{N}$ is a nucleation rate, $\nu_{0}$ a reference rate, *k* the Boltzmann constant, *T* the temperature and $W_{m}$, as given by Equation (73), appears as an energy barrier for the nucleation process [19]. Careful experiments on wood and fiberglass samples confirmed the lifetime vs. applied stress dependence $ln\;t_{N}∼\sigma^{-4}$ implied by Equation (74) [31], suggesting that crack growth is indeed a thermally-activated phenomenon. However, under realistic stress and temperature conditions, Equation (74) predicts exceedingly large nucleation times $t_{N}$, which points rather to irrelevance of the mechanism. The conundrum can be solved by assuming the presence of pre-existing defects, whose existence eases crack growth by lowering the energy barrier. Indeed, thermal fluctuations need only to augment a pre-existing defect of radius $l_{0}$ to the critical value $l_{c}$, rather than create a crack of radius $l_{c}$ from scratch. The energy barrier is consequently $W_{m}-W_{0}$, where:

(75) $$W_{0}=2\gamma \pi l_{0}^{2}-\frac{2\pi l_{0}^{3}\sigma^{2}}{3E}$$

is positive for $0<l_{0}<l_{c}$. The nucleation time $t_{N}$ therefore becomes:

(76) $$t_{N}=\nu_{0}^{-1}exp\left(\frac{2\pi }{kT}\left(\frac{4\gamma^{3}E^{2}}{3\sigma^{4}}+\frac{\sigma^{2}l_{0}^{3}}{3E}-\gamma l_{0}^{2}\right)\right),$$

which can be shown to take realistic values at room temperature, while not altering the correct lifetime vs. stress dependence [20]. Both ab initio and molecular dynamics simulations further confirm that microcracks may be created from thermally activated fluctuations of the atomic order in diamond or silicon crystals otherwise perfect [32,33,34]. Such crystal defects were shown to be at the origin of the brown coloration of diamonds [33]. In silicon, the nucleation of microcracks could be responsible for the strong chemical reactivity and electrical activity in the wake of moving dislocations [34]. Molecular dynamics simulations further suggest that microcracks may be formed in a perfect two-dimensional elastic crystal in tension at high strain rate, in a direction normal to the tensile axis, as a result of a competition between stress relaxation and atomic bond stretching [32]. Note that a common feature of microcrack nucleation in diamond and silicon is the concomitant nucleation of dislocation loops.

Despite successful predictions regarding the sample lifetime under stress, the thermal activation model for crack initiation does not provide the dynamics of the microcrack size *l*. Assuming a modified Langevin-type dynamics $\dot{l}=-M\partial W/\partial l+\eta \left(t\right)$ where *M* is a phenomenological mobility constant and η(t) a thermal noise contribution, [35] arrives at a lifetime vs. applied stress dependence at variance with the experimental data [31], which tends to rule out diffusive dynamics. Alternatively, by describing microcracks as disconnection loops, the present continuous modeling scheme provides a natural dynamics for crack growth, governed by the transport law (66) appended with the mobility relationship (68) between the driving force and the disconnection velocity. In order to investigate microcrack nucleation and growth in a simple setting by using this framework and in analogy with the calculations of [32], let us consider a sample loaded in simple tension at constant driving strain rate $\dot{\epsilon }>0$ along the loading direction $e_{2}$, with half-width *L* in the transverse direction $e_{1}$. Plane strain parallel to the plane $\left(e_{1},e_{2}\right)$ is assumed. Again, the material response is taken to be isotropic linear elastic, characterized by Young’s modulus *E* and Poisson’s ratio ν. The sample is initially free of cracks, but we look for conditions possibly allowing the nucleation of edge disconnections $\beta_{23}\left(x_{1},t\right)$ with transverse velocity $V=V_{1}e_{1}$ with respect to the material, i.e., for conditions possibly allowing Mode II crack nucleation and growth. For such disconnections, the transport Equation (54) reduces to:

(77) $$\frac{\partial \beta }{\partial t}+\frac{\partial }{\partial x}\left(\beta \left(v+V\right)\right)=0,$$

when the material velocity $v=v_{1}e_{1}+v_{2}e_{2}$ with respect to the reference frame is accounted for, and where the indices in α,v,V and *x* have been omitted. For the sake of simplicity of the model, it is provisionally assumed that the edge disconnection velocity *V* with respect to the material is:

(78) $$\begin{array}{ccc} V & = & V_{0}\frac{\beta }{\left|\beta \right|}=-V_{0}\;sgn\left(\beta \right),\;\beta \neq 0 \end{array}$$

(79) $$\begin{array}{ccc} V & = & 0,\;\beta =0, \end{array}$$

where $V_{0}$ is a constant velocity. Thus, no account is made of effects of the stress state on the disconnection velocity. It is then straightforward to show that the homogeneous distribution: $\forall \left(x,t\right),\;\beta_{0}\left(x,t\right)=\beta_{0}\left(t\right)=0$ with $\epsilon =\dot{\epsilon }t$ is solution to Equation (77). Clearly, this fundamental solution reflects the continuous deformation of a perfect elastic crystal without cracks. To analyze the stability of this solution, we look for inhomogeneous solutions β(x,t) to Equation (77) built by adding an inhomogeneous perturbation δβ to $\beta_{0}\left(t\right)$: $\beta \left(x,t\right)=\beta_{0}\left(t\right)+\delta \beta \left(x,t\right)$. With the above Griffith–Pomeau analysis in mind, such perturbations may be thought of as arising from thermal fluctuations of the atomic order in the lattice. In the homogeneous state: $\beta_{0}\left(t\right)=0$, and the associated velocity is V=0 according to relation (79). The disconnection perturbation δβ is necessarily non-zero and the sign of the associated velocity $\pm V_{0}$ is opposed to its sign, according to Equation (78). Substituting the inhomogeneous distribution $\beta \left(x,t\right)=\beta_{0}\left(t\right)+\delta \beta \left(x,t\right)$ into Equation (77) then leads to the evolution equation for δβ:

(80) $$\frac{\partial \delta \beta }{\partial t}+\frac{\partial }{\partial x}\left(\left(v+V\right)\delta \beta \right)=0.$$

The inhomogeneous perturbations δβ are looked for in the spectral form:

(81) $$\begin{array}{ccc} \delta \beta_{k} & = & \delta {\hat{\beta }}_{k}e^{\lambda_{k}t}e^{i\xi_{k}x} \end{array}$$

where $\left(\delta {\hat{\beta }}_{k},\lambda_{k},\xi_{k}\right)$ are constants. $\xi_{k}=2\pi k/L$ is to be interpreted as the perturbation wave number per unit length. $\lambda_{k}$ is a (possibly complex) perturbation growth rate and $\delta {\hat{\beta }}_{k}$ an initial lattice defect. Using this development in Equation (80), it is found that:

(82) $$(\lambda_{k}+i\xi_{k}\left(v+V\right)-\nu \dot{\epsilon })\delta \beta =0.$$

Thus, non-vanishing perturbations δβ are allowed when the eigenvalue $\lambda_{k}$ is:

(83) $$\lambda_{k}=\nu \dot{\epsilon }-i\xi_{k}\left(v+V\right).$$

The real part of $\lambda_{k}$: $Re\left(\lambda_{k}\right)=\nu \dot{\epsilon }$ is the growth-rate of the perturbation. It is positive in tension ($\dot{\epsilon }>0$), irrespective of the wave number *k*, implying that all perturbation modes grow and that the fundamental state $\beta_{0}\left(t\right)$ is unstable. In addition, the perturbation growth rate increases with the strain rate $\dot{\epsilon }$, and this result holds true for all initial amplitudes $\delta {\hat{\beta }}_{k}$. The resulting disconnection density modes are in the form:

(84) $$\begin{array}{c} \beta_{k}\left(x,t\right)=\delta {\hat{\beta }}_{k}e^{\nu \epsilon }e^{-i\xi_{k}\left(-\nu \dot{\epsilon }x+V\right)t}e^{i\xi_{k}x} \end{array}$$

where $\epsilon =\dot{\epsilon }t$ denotes the compatible tensile strain achieved since the initial time and $v=-\nu \dot{\epsilon }x$ is the transverse material velocity. Re-arranging and extracting the real part, it is found that:

(85) $$\beta_{k}\left(x,t\right)=\delta {\hat{\beta }}_{k}e^{\nu \epsilon }cos\;2\pi k\frac{X-Vt}{L},$$

where X=(1+νϵ)x. The meaning of Equation (85) is that a disconnection density spectral component emerges from arbitrarily small thermal fluctuations in the tension of the sample and that the nucleated disconnections travel away from the material point *X* at velocity $\pm V_{0}$ with respect to the material. Since positive and negative values of $\delta {\hat{\beta }}_{k}$ are equally admissible, dipoles of edge disconnections are actually nucleated. The latter are the traces along the *x*-axis of disconnection loops representing slit cracks in a three-dimensional setting, and the outward motion of the edge dipoles at velocity $\pm V_{0}$ reflects the propagation of these cracks. Further, nucleation may be localized in a small area patch by linearly combining all the spectral modes, because all wave numbers *k* are equally admissible:

(86) $$\beta \left(x,t\right)=e^{\nu \epsilon }\sum_{k=1}^{\infty }\delta {\hat{\beta }}_{k}cos\;2\pi k\frac{X-Vt}{L}.$$

The origin for disconnection growth can be traced to shrinkage of the circuit *C* of Equation (14) due to Poisson’s effect in tension and to conservation of the COD f in this motion, in the absence of any incoming/outgoing disconnection flux. Thus, thermal fluctuations of the atomic arrangement may be converted into expanding disconnection loops if the applied loads provide adequate conditions.

In order to account for the relaxation of the stress field accompanying crack nucleation, we now evaluate the incompatible strain field arising from the disconnection distribution (86) in a unidimensional approximation. The incompatible distortion satisfies Equation (60), which reduces here to $U_{22,1}^{⊥}=\epsilon_{22,1}^{⊥}=-\beta_{23}$ or, using Equation (86):

(87) $$\frac{\partial \epsilon^{⊥}}{\partial x}=-e^{\nu \epsilon }\sum_{k=1}^{+\infty }\delta {\hat{\beta }}_{k}cos\;2\pi k\frac{X-Vt}{L}.$$

Assuming all initial defects to be identical: $\forall k,\;\delta {\hat{\beta }}_{k}=\delta \hat{\beta }$, we find:

(88) $$\epsilon^{⊥}=-\frac{L\delta \hat{\beta }}{2\pi }\frac{e^{\nu \epsilon }}{1+\nu \epsilon }\sum_{k=1}^{+\infty }\frac{sin\;2\pi k\frac{X-Vt}{L}}{k}=-L\delta \hat{\beta }\frac{e^{\nu \epsilon }}{2(1+\nu \epsilon )}\left(\frac{1}{2}-\frac{X-Vt}{L}\right),$$

provided X−Vt<L (see Item 1.441.1 in [36]). A first-order approximation of $\epsilon^{⊥}$ valid in the close vicinity of the nucleation point is therefore:

(89) $$\epsilon^{⊥}≅-\delta \beta_{0}\frac{e^{\nu \epsilon }}{1+\nu \epsilon }$$

where $\delta \beta_{0}=L\delta \hat{\beta }/4$ is an initial nondimensional disconnection density. For linear elasticity, the tensile stress accounting for both the compatible and incompatible strain fields is therefore (see Equation (63)):

(90) $$\sigma =E(\epsilon -\delta \beta_{0}\frac{e^{\nu \epsilon }}{1+\nu \epsilon }).$$

Clearly, σ first increases with strain up to a maximum and eventually drops down to zero. The elastic energy density ${\left(\epsilon^{⊥}\right)}^{2}/2E$ resulting from the incompatible elastic strain $\epsilon^{⊥}$ appears as the translation in the present continuous analysis of the surface energy of Griffith’s model, while the elastic energy density $\epsilon^{2}/2E$ in the latter is actually the energy arising from the compatible elastic strain energy in the former. Thus, the condition for the maximum tensile stress in the present model corresponds to Griffith’s criterion. In addition, the present analysis yields a dynamics of crack growth, which develops by transport from the less-than-critical thermally-activated fluctuations of the atomic order. If, however, it is now demanded that the dislocation velocity $V_{0}$ decrease with the tensile stress and vanish below some positive threshold stress according to some Griffith-type criterion, then crack growth eventually stops and the tensile stress σ remains positive. However, the present unidimensional analysis cannot capture this situation, for which numerical simulations are necessary. The present conclusions are very similar to the results of the atomistic simulations [32,33,34], although the methods used are clearly different. It must be noticed further that similar conclusions may be obtained from a parallel treatment of the nonlinear dislocation transport Equation (57), implying that there is an equal chance to nucleate edge dislocations loops in similar numbers in the process. This remark might help to explain the concomitant presence of microcracks and dislocation loops mentioned above.

## 8. Application to DIC Methods and Strain Localization-Induced Fracture in Al-Cu-Li Alloys

In this section, we propose that our theoretical framework can be of practical use to complement experimental analysis of links between strain localization and fracture initiation in materials, in terms of strain incompatibility and associated disconnection densities. In particular, targeted applications are DIC-based methods, including standard techniques that use painting speckles [21], as well as more complex three-dimensional or high-resolution techniques that can use small grids or precipitates [22,23,37,38]. The idea is that the evolution of spatial gradients of the material distortion field, which are involved in the estimation of the disconnection densities, can help in predicting the occurrence of cracks in relation to strain localization. We expect in particular that our framework can help in establishing links between strain/strain rate distributions and corresponding crack modes and profiles. Alternatively, it may also be used to complement experimentally validated damage/fracture models. In this work, we propose to apply our disconnection framework to standard two-dimensional DIC data obtained during tensile quasi-static loading of Al-Cu-Li alloys. As aforementioned, these alloys are prone to complex interactions between localized plasticity and damage evolution leading to failure. This study can be considered as a benchmark for our modeling framework. Before describing the association of crack nucleation/characteristics with different components of disconnection densities, we will first explain below the experiments performed, the standard DIC technique used, and the procedure involved in the derivation of disconnection density maps from DIC data.

### 8.1. DIC Setup and Estimation of Disconnection Densities from DIC Data

**Figure 2:**
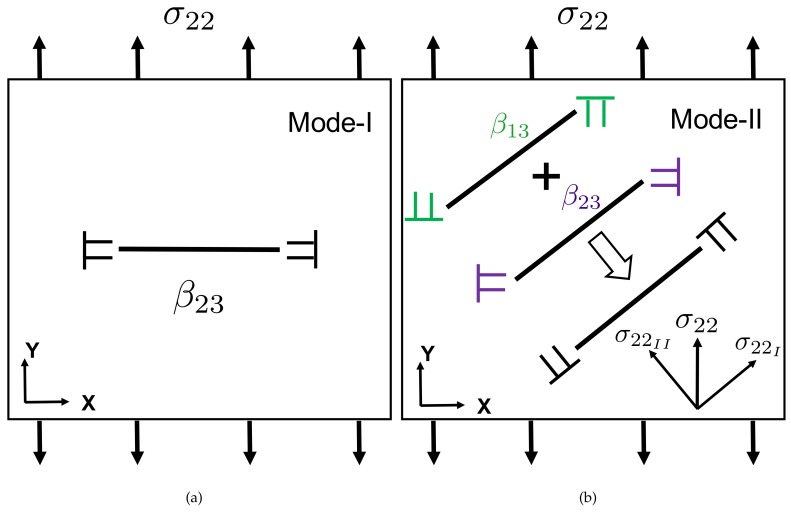
Representation of different fracture modes in terms of disconnection dipoles and loading direction: (**a**) Fracture Mode I; (**b**) Fracture Mode II.

**Figure 3:**
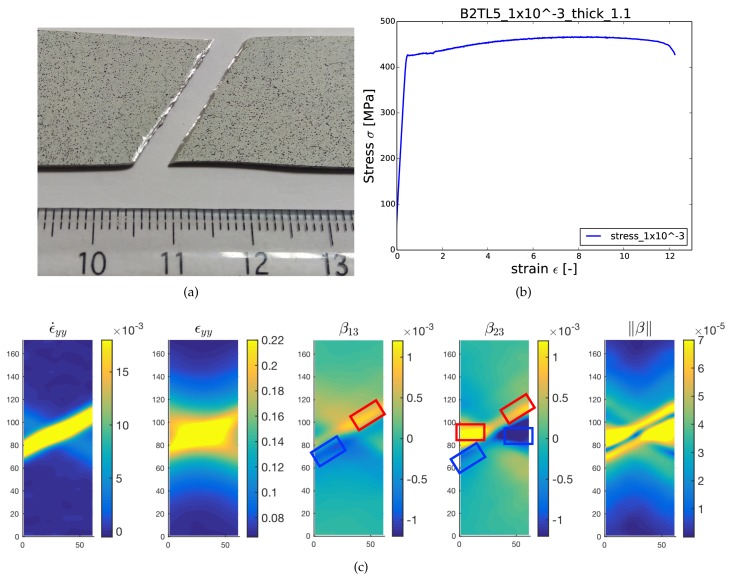
Fracture characteristics of the specimen B2TL5 broken in mixed mode (under uniaxial tension): (**a**) slanted fracture of the sample B2TL5; (**b**) stress/strain curve; (**c**) DIC maps for strain (no unit), strain rate (/s) and disconnection density fields (arbitrary units) evaluated just before the fracture.

Digital image correlation (DIC) is a non-contacting optical technique for the full-field deformation measurements [39,40]. Standard DIC methods, such as the one used in the present work, allow deriving the strain field on a sample surface as per following the evolution of a painting speckle pattern deposited on the sample surface by spray. Such methods have several advantages, such as no particular surface preparation needed, mostly implemented in situ and minimum setup requirement. In the present case, DIC has been applied with the objective to measure the local strain fields, not only all along the deformation test, but also and more importantly just before material failure. DIC methods devoted to fracture already exist in the literature, where special care is to be taken to treat the discontinuity of displacement field at crack faces [41,42,43,44]. The method we propose here rather consists of integrating the evolution with strain of some skewed disconnection density components, as detailed below, up to failure, but not at the time of failure. Eventually, we can analyze if the skewed disconnection densities correlate with the fracture of the sample and if they can help in interpreting/anticipating fracture profiles and crack modes in relation to strain/strain rate distributions and evolutions. The 2D DIC setup used for the purpose consists of an imaging source CCD camera (AVT Pike F-421B) with a resolution equal to 2048×2048 pixels, a Myutron lens with a 50-mm focal length to control focus, zoom and f-stop and an artificial speckle pattern on the specimen to be examined. Speckle patterns employed can be seen in the Figure 2 and Figure 3. Digital images were captured at a frame rate of 5 to 10 Hz (depending on the strain rate) during the tension tests, and thereafter, post-processing of the images was done using VIC2D commercial software from correlated solutions in order to get the pixel by pixel strain, strain rate and displacement maps at the specimen surface.

As an extended use of DIC, strain rate maps are used as an input to evaluate the distortion rate (symmetric part is strain rate) and thereby some skewed disconnection density component rates, during loading and just before crack nucleation in ductile materials. The compatibility condition (Equation (9)) holds until the onset of a displacement discontinuity; this is the point where a non-vanishing disconnection density field is expected to emerge in accordance with Equation (15). Our intent is to demonstrate the ability of such an approach to characterize the nucleation of a crack. To this end, we consider a flat surface of a dog bone-shaped tensile specimen loaded along direction $e_{2}$, in the plane $\left(e_{1},e_{2}\right)$ of the orthonormal frame $\left(e_{1},e_{2},e_{3}\right)$. From the DIC recorded data, we can use the in-plane distortion rate fields $\left({\dot{U}}_{11},{\dot{U}}_{12},{\dot{U}}_{21},{\dot{U}}_{22}\right)$ of the surface on a grid (if shear distortion rates are not available they can be evaluated from spatial derivatives of displacement rates), from which the rate of disconnection densities ${\dot{\beta }}_{13}={\dot{U}}_{11,2}-{\dot{U}}_{12,1}$ and ${\dot{\beta }}_{23}={\dot{U}}_{21,2}-{\dot{U}}_{22,1}$ may be numerically evaluated. We did it with a very simple MATLAB script that uses DIC mat files generated by VIC2D software. In the absence of a displacement rate discontinuity, ${\dot{\beta }}_{13}={\dot{\beta }}_{23}=0$, according to the rate form of Equations (8) and (9). However, to anticipate and detect crack nucleation, i.e., the advent of a non-zero disconnection density distribution, we slightly skew the curl operator in Equation (15) and define instead the modified rate of disconnection densities ${\dot{\beta }}_{13}^{\prime }=\left(1+\epsilon \right){\dot{U}}_{11,2}-\left(1-\epsilon \right){\dot{U}}_{12,1}$ and ${\dot{\beta }}_{23}^{\prime }=\left(1+\epsilon \right){\dot{U}}_{21,2}-\left(1-\epsilon \right){\dot{U}}_{22,1}$, where ϵ<<1. As a result, the skewed-disconnection densities $\left(\beta_{13}^{\prime },\beta_{23}^{\prime }\right)$, integrated over time during the deformation of the material, are non-vanishing even if $\beta_{13}=\beta_{23}=0$. The amplitude of disconnection densities (to avoid the confusion, skewed disconnection densities $\beta_{13}^{\prime }$ and $\beta_{23}^{\prime }$ are presented as disconnection densities $\beta_{13}$ and $\beta_{23}$ itself), as well as their sign both depend on the artificial parameter ϵ, and no quantitative conclusion will be drawn in this paper (strong uncertainty in displacement and strain fields as obtained from the DIC analysis also prevents from providing a reliable quantitative description of fracture). However, we can still expect that monitoring the variations of the disconnection densities will provide very valuable qualitative information on the emergence of a crack as the sample deformation proceeds. Furthermore, we can expect that comparison of relative weights and distributions of available disconnection density components can provide information on emerging crack modes (only Mode I and Mode II in 2D).

To test our extended DIC post-treatment analysis, we used tensile specimens extracted from Al-Cu-Li AA-2198-T8 alloys. Experimental data on such alloys can be found in the literature [22,45]. This material was industrially processed into sheets of thickness 2.5mm. The processing routes involved rolling and several thermo-mechanical heat-treatments leading to a multi-layered morphological texture with grains strongly elongated in the rolling direction L. Machining may have been additionally used to reduce further the sheet thickness to 1.1mm from the initial thickness, while keeping the grain thickness unchanged. Thus, the thickness, width and gauge-length of the tested specimens were (2.5mm,20mm,120mm) and (1.1mm,20mm,120mm). In each case, samples have been loaded in both the rolling L and transverse T direction. Failure of the samples occurred while the load was decreasing after the nominal ultimate tensile strength (UTS), and moderate necking was observed, particularly when loading in the T direction.

### 8.2. Analysis of Sample Fracture Using Disconnection Densities

**Figure 4:**
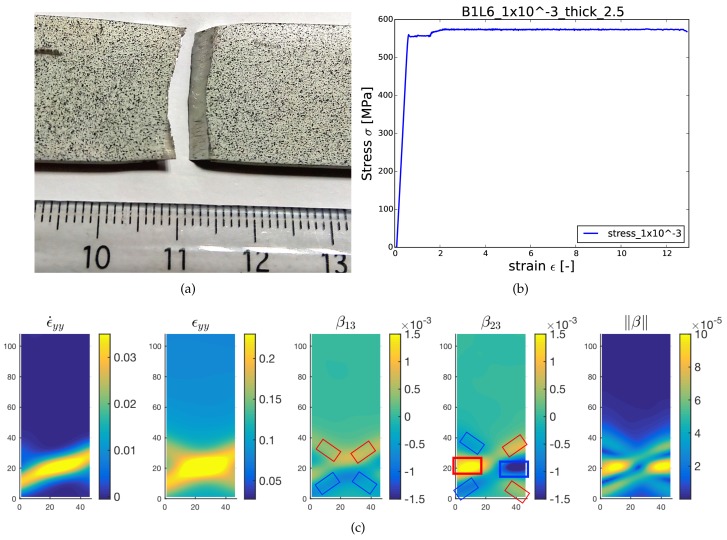
Fracture characterization of the specimen B1L6 predominantly failed in Mode I: (**a**) fracture pattern of broken specimen; (**b**) stress/strain curve; (**c**) digital image correlation (DIC) maps for strain (no unit), strain rate (/s) and disconnection density fields (arbitrary units) evaluated just before the fracture.

Predominantly, the specimens fractured in a mixed mode configuration (both Mode I and Mode II are present). Mixed mode resulted in a crack along slanted bands at an angle with the loading direction. In a first case shown in this paper, the fracture band is rotated around both directions normal to the loading direction (Figure 3). In a second case also shown in the paper, the fracture band is only inclined in the thickness direction (Figure 4). Although it is a mixed mode, we refer to that last case as a Mode I dominated fracture, as compared to the first case. Indeed, the interpretation of this inclination in the thickness direction, in terms of disconnection densities, would require 3D DIC data, which is not available in the present work. As such, with the 2D analysis presented hereafter, we can only describe the Mode I fracture component in the second sample. For this particular alloy and the tensile tests performed here, the crack initiation mechanism was not investigated. Damage evolution and eventual fracture might be the result of strain accumulation induced void growth [22]. Void nucleation may occur at micrometric intermetallic particles after sufficient strain [23]. Grain boundary decohesion might also be involved because of the strongly rolled microstructure [45]. Before starting to explore the connection between fracture nucleation/characteristics and disconnection theory, it is necessary to understand how different fracture modes can be represented in terms of disconnection density dipoles and loading direction [13]. Following the dislocation-based fracture mechanics framework [11], one can characterize cracks in the DIC observation domain in terms of disconnection density dipoles, which manifest opening and closing of a full crack. When the crack face is perpendicular to the tension axis, it is known as a Mode I crack and the latter can be represented a dipole of $\beta_{23}$ components with the disconnection densities of opposite sign being located at the crack tips and delimiting the crack. This case is well described in a recent paper [13]. This case is shown in Figure 2a. However, if such a dipole was vertical in the figure and the sample was loaded in shear (in plane shear), it would correspond to a Mode II crack (we are leaving out the Mode III for now as we have only access to 2D data). In the present tension tests, Mode II cracking can still be observed as a resultant of combined $\beta_{23}$ and $\beta_{13}$ disconnection dipoles, as shown in Figure 2b. We have analyzed several specimens, which were fractured fortunately in the DIC observation window, and among them, two representative cases are discussed here.

### 8.3. Mixed Mode Fractured Specimen

This specimen (B2TL5) was tested in the uniaxial tension along transverse direction at a constant strain rate $1\times 10^{-3}/\sec$, the global stress strain curve being presented in Figure 3b. We are only interested in the characteristics of the fracture and therefore, DIC maps are presented only for the time frame just before the fracture took place. Figure 3 shows the fracture pattern of the specimen and associated DIC maps for strain rate, strain and disconnection components fetched from the DIC post-treatment analysis. As shown in Figure 3a, the specimen was broken in a slanted manner which can be referred to as mixed mode fracture and can be explained on the basis of maps presented in Figure 3c. Both fracture modes described in Figure 2 (regarding disconnection distribution resulting in Mode I and Mode II) fit well for the β distribution obtained in the present fracture case. Indeed one can see first an horizontal dipole of $\beta_{23}$, which corresponds to Mode I, and also an inclined dipole made of both $\beta_{13}$ and $\beta_{23}$, which corresponds to Mode II fracture (dipoles are represented by blue and red boxes on β distribution maps). Looking at the norm of disconnection density tensor ||β||, one can clearly anticipate that the upcoming slanted mixed mode fracture of the sample. Although this slanted profile is hardly visible in the accumulated strain distribution, it can be clearly identified in the disconnection density maps, which correlate also well with the strain rate map.

### 8.4. Mode I Dominated Fractured Specimen

The specimen (B1L6) was tested in uniaxial tension along rolling direction at a constant strain rate $1\times 10^{-3}/\sec$, the global stress strain curve being presented in Figure 4b. As mentioned earlier, due to the sole interest in fracture, DIC maps presented here are only associated with the time frame just before the actual fracture took place. Figure 4 demonstrates fracture characteristics of the broken specimen, including fracture pattern and associated DIC maps for strain rate, strain and disconnection density calculated from the DIC analysis. Figure 4a shows that the specimen was broken perpendicular to the loading direction, which can be referred to as Mode I-dominated fracture. As a general observation in all our tests not shown here, it is found that tension tests in transverse direction resulted primarily in mixed mode fracture whereas rolling direction favored the fracture dominated by Mode I with few exceptions. The fracture observed for the B1L6 sample can be explained well with the help of β distribution maps shown in Figure 4c. Analogous to B2TL5, dipoles of $\beta_{13}$ and $\beta_{23}$ are also visible in this case (they are again marked with red and blue boxes on β distribution maps). However, the horizontal dipole of $\beta_{23}$ reflecting Mode I is observed to be much stronger in magnitude than the dipoles manifesting Mode II. In this sample, two inclined dipoles of lower magnitude and corresponding to Mode II fracture can be seen in the maps (blue and red boxes with thinner lines than boxes corresponding to Mode I horizontal dipole). They are disposed in a symmetric manner with respect to the sample and loading direction, as can be seen in the norm of the disconnection density tensor. The latter norm clearly suggests that Mode I fracture will be predominant for this sample. It also suggests that the inclined dipoles corresponding to Mode II are canceling out because of symmetry. Similarly to the B2TL5 sample, the fracture pattern is in good agreement with the distributions of disconnection densities. Here however, while the fracture profile shows also good correlation to the strain rate map for the B2TL5 sample, it does not really fit for the B1L6 sample, and the disconnection density analysis seems to be the most predictive quantity.

## 9. Conclusions

We proposed a geometrically nonlinear theory of coupled plasticity and fracture in crystalline materials dealing with the statics and dynamics of smooth crystal defect density fields, namely dislocation and disconnection densities, as introduced in [13]. While dislocations are line defects terminating surfaces across which the plastic displacement field encounters a discontinuity, disconnections are defined as line defects terminating surfaces across which the total displacement faces a discontinuity, thus reflecting fracture of the body. The paper builds on the duality existing between the discontinuity of the displacement field and the incompatibility (non-integrability) of the associated smooth distortion field to introduce a field theory of fracture. While the incompatibility of the plastic distortion lends its meaning to the tensorial density of dislocations α, the disconnection density tensor β acquires its topological significance from the incompatibility of the total distortion. When the latter remains compatible, i.e., when it reduces to a gradient tensor, continuity of the body is maintained, and the present theory reduces to the mechanics of dislocation fields [46]. The achievements of the Peierls model in elucidating basic dislocation physics testifies to the appeal of field descriptions for the analysis of line defects. Such attractiveness lies in that, when viewed at a sufficiently small scale, line defects are better described by a suitably-localized smooth density field than by a singularity. Smoothness of the description is not only desirable from the point of view of mathematical analysis and numerical computation, but also because it allows coping with core properties. Beyond regularizing the description of individual cracks, disconnections may also be useful, if a larger resolution length scale is chosen, in describing damage distributed throughout the body.

Further interest in considering disconnection density fields for fracture modeling arises from their conservation laws, which are unquestionable from a kinematic point of view and, hence, provide a natural framework for their dynamics and consequently for crack growth. This feature is used in the present paper to introduce a set of partial differential equations (PDEs) for the dynamics of the compatible displacement and dislocation/disconnection density fields, reflecting respectively dislocation motion and crack growth. A prominent property of the theory is therefore that energy dissipation may occur concurrently through crack growth and dislocation motion, both in the bulk of the body and along the crack surfaces. The Peach–Koehler force on dislocations, as well as the driving forces for disconnection motion and crack surface separation, are found as dual quantities to the respective defect velocities in the dissipation. Eventual constitutive choices include a Griffith-type criterion for disconnection transport (fracture growth), as well as a cohesive-type behavior for the crack opening displacement. While being based on PDEs in the set of variables involved, i.e., the compatible components of the total displacement, the dislocation and disconnection densities, the present theory is nonlocal in space and time in the standard variables of conventional continuum mechanics. Thus, it provides nonlocal generalization of the latter, leading to well-posed problems in dislocation and fracture dynamics. Generating approximate solutions to the present theory, while being non-trivial, leads to finite-element or spectral FFT-based implementations similar to those already employed in the pure dislocation dynamics case in [29,47] and [48,49]. However, a fundamental difference between disconnections and dislocations lies in that the stress field of disconnections is dependent on the actual location of the surface of displacement discontinuity, because the traction vector vanishes along this surface, whereas the stress field of dislocations does not depend on this position, but only on that of the dislocation line. Hence, the history of the surface of discontinuity must be kept track of in implementing disconnection dynamics.

As an illustration of the effects of nonlinearity, thermally-activated crack nucleation and growth were evidenced in this paper by using the disconnection density concept in elastic-brittle materials. It is revealing that the elastic energy density arising from the incompatible elastic strain field in the present theory plays the role of the surface energy density associated with atomic bond breaking in Griffith’s theory of crack nucleation, while the elastic energy density in the latter is actually the energy density arising from the compatible elastic strain field in the former. Here, crack nucleation appears as a natural consequence of the geometrical nonlinearity of transport: an initial whiff of disconnection density due to thermal agitation of the atomic lattice may grow if the deformation process tends to shrink the surrounding material, because the COD content of elementary patches must be conserved. Eventually, the spatio-temporal dynamics of the disconnection density fluctuation is also governed by transport, which results in further propagation of the nascent crack.

Finally, the DIC disconnection density-based analysis of tensile tests performed on ductile Al-Cu-Li samples not only validates the disconnection density framework, but it also shows that disconnection densities can help to anticipate and to describe crack nucleation and morphology in relation to strain and strain rate distribution and evolution. For some samples tested in this work, we observed fracture profiles that were in good agreement with disconnection density maps, but less with strain and strain rate maps. Further work on Al-Cu-Li alloys, consisting of using disconnection densities for modeling strain localization induced fracture, is under progress. We also think that our DIC disconnection extension can provide a valuable and complementary analysis of fracture. Importantly, it is very easy to implement in a DIC analysis code, and the methodology could be applied to recent high-resolution DIC techniques [37,38] and to 3D DIC data [22,23] for a full 3D description of cracks and a fine resolution scale analysis of fracture in relation to strain localization.
